# Identifying Medicine Shortages With the Twitter Social Network: Retrospective Observational Study

**DOI:** 10.2196/51317

**Published:** 2024-08-06

**Authors:** Doerine J Postma, Magali L A Heijkoop, Peter A G M De Smet, Kim Notenboom, Hubert G M Leufkens, Aukje K Mantel-Teeuwisse

**Affiliations:** 1 Division of Pharmacoepidemiology and Clinical Pharmacology Utrecht Institute for Pharmaceutical Sciences (UIPS) Utrecht University Utrecht Netherlands; 2 Royal Dutch Pharmacists Association The Hague Netherlands; 3 Departments of IQ Healthcare and Clinical Pharmacy Radboud Institute for Health Sciences Radboud University Medical Centre Nijmegen Netherlands; 4 Dutch Medicines Evaluation Board Utrecht Netherlands

**Keywords:** medicine shortages, signal detection, social media, Twitter social network, drug shortage, Twitter

## Abstract

**Background:**

Early identification is critical for mitigating the impact of medicine shortages on patients. The internet, specifically social media, is an emerging source of health data.

**Objective:**

This study aimed to explore whether a routine analysis of data from the Twitter social network can detect signals of a medicine shortage and serve as an early warning system and, if so, for which medicines or patient groups.

**Methods:**

Medicine shortages between January 31 and December 1, 2019, were collected from the Dutch pharmacists’ society’s national catalog Royal Dutch Pharmacists Association (KNMP) Farmanco. Posts on these shortages were collected by searching for the name, the active pharmaceutical ingredient, or the first word of the brand name of the medicines in shortage. Posts were then selected based on relevant keywords that potentially indicated a shortage and the percentage of shortages with at least 1 post was calculated. The first posts per shortage were analyzed for their timing (median number of days, including the IQR) versus the national catalog, also stratified by disease and medicine characteristics. The content of the first post per shortage was analyzed descriptively for its reporting stakeholder and the nature of the post.

**Results:**

Of the 341 medicine shortages, 102 (29.9%) were mentioned on Twitter. Of these 102 shortages, 18 (5.3% of the total) were mentioned prior to or simultaneous to publication by KNMP Farmanco. Only 4 (1.2%) of these were mentioned on Twitter more than 14 days before. On average, posts were published with a median delay of 37 (IQR 7-81) days to publication by KNMP Farmanco. Shortages mentioned on Twitter affected a greater number of patients and lasted longer than those that were not mentioned. We could not conclusively relate either the presence or absence on Twitter to a disease area or route of administration of the medicine in shortage. The first posts on the 102 shortages were mainly published by patients (n=51, 50.0%) and health care professionals (n=46, 45.1%). We identified 8 categories of nature of content. Sharing personal experience (n=44, 43.1%) was the most common category.

**Conclusions:**

The Twitter social network is not a suitable early warning system for medicine shortages. Twitter primarily echoes already-known information rather than spreads new information. However, Twitter or potentially any other social media platform provides the opportunity for future qualitative research in the increasingly important field of medicine shortages that investigates how a larger population of patients is affected by shortages.

## Introduction

The continuous supply of quality-assured, safe, effective, and affordable medicines is one of the building blocks of a well-functioning health system [1]. European legislation stipulates that marketing authorization holders (MAHs) should ensure appropriate and continuous supplies so that the needs of patients in the member state in question are met [2]. Despite this requirement, access to medicines and vaccines is a concern, because of the persistent problems of shortages and stockouts [3]. With the COVID-19 pandemic, this reality was even more pronounced as the problem of shortages of essential medicines worsened [4,5]. It brought the long-standing vulnerability of the medical product supply chain into sharp focus [6].

For mitigating the impact of medicine shortages on patients, early identification is considered critical. It allows for early triaging, assessment, and coordination of medicine shortages to prevent them or minimize their impact [7,8]. The World Health Organization urged member states to implement effective notification systems that allow measures to avoid medicines and vaccine shortages [1].

MAHs in Europe are required to notify competent national authorities of expected supply interruptions 2 months in advance and of unexpected interruptions as soon as possible [2]. Competent authorities are recommended to communicate any nationwide shortage, depending on available resources and the communication needs within their territories [9]. In the Netherlands, competent authorities publish details, actions taken, and advice on the shortages of critical products for health care professionals. Products are considered critical if they meet 2 criteria. First, they are part of the treatment or prevention of a life-threatening or irreversibly progressive disease, and second, there is insufficient availability of equivalent alternatives [10,11]. The publication of all notifications submitted by MAHs to the Dutch authorities would not be meaningful because many of these notifications concern the risk of a supply disruption, but the supply is often unaffected. Most supply disruptions also concern a shortage of a product for which generic substitution is available and this substitution is considered to have a low impact on patients [12].

Health care professionals, especially pharmacists, are confronted daily with supply disruptions and seek information on and solutions for critical and noncritical shortages. In addition to the information authorities publish on shortages, many (European) pharmacists’ societies publish national catalogs of identified shortages when a pharmacy cannot supply a requested product to patients. However, these catalogs are often published too late to mitigate the shortages’ impact [13,14].

Patients may be directly impacted by shortages; they may, for example, receive an alternative therapy or face additional costs [15]. The research on the impact of medicine shortages on patients has been limited to qualitative research on a small number of patients [16]. They could potentially also play a role in the early identification of shortages, but this has not been studied yet. In pharmacovigilance, patients—or the general public—have often flagged medicine-related issues at an early stage, specifically adverse drug reactions [17-19]. How the general public learns about diseases and medicinal products has dramatically changed due to the internet [20,21]. Social media, in particular, are an emerging source of health data [22]. Social media platforms, such as Instagram (Meta Platforms), Facebook (Meta Platforms), and the Twitter social network (X Corp; recently transformed to X), allow users not only to inform themselves but also to reach a general audience of other internet or platform users. Despite the biases that arise when using social media in research [23], we believe that social media platforms should be studied as a possible source of health information.

In health research, the most commonly studied social media platforms are Facebook and Twitter. Studies have shown that Facebook has been primarily used for recruitment, whereas Twitter has been used for content analysis [24], early warning [25], and event detection [26]. Recent examples of Twitter as an early warning system include warnings for new COVID-19 outbreaks [26] and safety warnings on medicinal products [27,28]. Twitter provides a unique big data source for public health researchers because of the concise messages, the real-time nature of its content, and the ease of searching and accessing publicly available information on it [25]. Twitter users post messages (“posts”—formerly known as tweets) with a maximum of 280 characters at the time of research, visible to anyone following the sender. With 2.8 million users in the Netherlands in 2019, Twitter was one of the largest social media platforms [29].

Since patients or the general public can potentially identify shortages and provide early insight into them, this study aimed to explore whether the routine analysis of Twitter data can be used as an additional early warning system for medicine shortages in the Netherlands and, if so, for which medicines or patient groups.

## Methods

### Data Source

We collected data on temporary and permanent medicine shortages from Royal Dutch Pharmacists Association (KNMP) Farmanco between January 1 and December 31, 2019, the year before the COVID-19 pandemic. In the Netherlands, KNMP Farmanco is the most complete data set of medicine shortages. The Dutch pharmacists’ society, KNMP, receives signals on possible shortages from Dutch pharmacy practice. The MAH validates these shortages and may also report shortages itself. KNMP Farmanco publishes data on its website within 24 hours of reports by pharmacists or others [30]. Information on the shortage (such as the product in shortage and the expected resolution date) is publicly available, but information on possible solutions for patients is restricted to health care professionals (mainly pharmacists).

On this website, a shortage is defined as the national unavailability for at least 2 weeks of an active pharmaceutical ingredient with at least 1 medicinal product for human use with marketing authorization. A period of fewer than 2 weeks of unavailability is likely to be mitigated by stock still present in the supply chain, as patients’ remaining supply, or as other pharmacies’ and wholesalers’ stock. Therefore, the KNMP chooses 2 weeks as the cutoff point [31]. The output file that we retrieved for shortages during the study period contained data on the medicine’s name, the active pharmaceutical ingredient, the dates the shortage was published and resolved, and possible alternative products for treating patients.

Data on the number and median age of patients affected by the shortages were obtained from the Dutch Foundation for Pharmaceutical Statistics (SFK) by analyzing the number of patients 12 months before the shortage. The SFK only has data on medicines dispensed in community and outpatient pharmacies. No data were available on products dispensed in hospital pharmacies.

We used posts from Twitter due to the advantage of the real-time nature of its content and the ease of searching and accessing publicly available information, despite not being the largest platform in the Netherlands (ranking after for example Facebook [29]). Posts on these shortages were retrieved from the Utrecht Data School. These posts were publicly available and published between January 1 and December 31, 2019. The output file contained data on the post ID, post text, user ID, date and time created, and the name of the medicine mentioned. When a post noted more than one medicine, we included this post separately for each medicine.

### Data Selection

A total of 389 medicine shortages occurred in the Netherlands in 2019. We decided only to match a post to a shortage if the post was published no more than 30 days before the shortage was published and no more than 30 days after the shortage ended, according to KNMP Farmanco. We thus included shortages between January 31 and December 1, 2019, resulting in 341 shortages for this study.

For these 341 shortages, Dutch posts were collected by searching for the names of the medicines in shortage: the Anatomical Therapeutic Chemical (ATC) name on the fifth level, which is the active pharmaceutical ingredients [32] or the first word of the brand name. For instance, a dermal product with betamethasone may be best known by its brand name Diprosole, and tablets with clopidogrel may be best known by the brand name Plavix. We excluded reposts. We did not account for misspellings and common wording for groups of medicines, since we were aiming to assess the use of Twitter data in routine analysis.

Posts were then selected based on relevant keywords that potentially indicated a shortage (Multimedia Appendix 1). These keywords were developed in 2 rounds. We initially had a set of 6 keywords (almost out of, available, discontinued, scarce, shortage, and supply) that we as researchers expected to be used. Since this set did not identify the major shortages we had expected, we took a different approach. We analyzed all (>10,000) posts on 20 pharmaceutical substances. Based on this screening, we determined the final set of 30 keywords (adding keywords such as delivery date, deficiency, lack of, missing, present, and stock; Multimedia Appendix 1) that we used for this paper.

Using this final set of keywords, we selected all posts for the 341 shortages in our study. A researcher (MLAH) assessed these posts to determine if the post was about a mentioned medicine shortage. The assessment was based on individual posts and if they were ambiguous, a series of connected posts (thread) and replies to the post were studied. Inconclusive posts were assessed by a second researcher (DJP), and consensus was reached following discussion.

### Data Analysis

#### Mentioned on the Twitter Social Network or Not

We characterized the products in shortage by their ATC class on the first level [32], according to the organ or the system on which they act. We compared the characteristics of the medicine shortages and the patients affected by these shortages, for shortages mentioned and not mentioned on Twitter. We used the Fisher test (exact if possible or Monte Carlo if necessary) for categorical data, and for continuous quantitative data, we used the Levene median test. *P* values <.05 were considered significant.

#### Timing of Posts

We analyzed the posts for their timing with the publication of shortages by KNMP Farmanco. If more than 1 post was published during the shortage, we selected the first post chronologically. If the difference in timing of the publications was 2 days or fewer, we considered the publications simultaneously since administrative processing (eg, reporting during weekends) can cause this difference. To determine differences in timing between stakeholder categories, we analyzed only stakeholder categories with more than 10 posts.

#### Content Analysis

We grouped the senders according to stakeholder category based on the sender’s name, the profession stated in his or her profile, and the content of the post message (Textbox 1). The content of the posts was also analyzed and categorized based on its nature. While the first posts served as the main analysis (Textbox 2), all posts were analyzed and categorized. A researcher (MLAH) categorized these posts. A second researcher (DJP) independently categorized inconclusive posts, and if necessary, consensus was reached following the discussion.

**Stakeholder categories.**

- Patient: post contains phrase such as “my medicine...”
- Health care professional: post contains phrase such as “my patients...” or profile states profession
- Media: profile or ID states name of media
- Others: profile or ID states name of pharmaceutical industry, government, or health insurers
- Unknown: insufficient information to determine the stakeholder

**Nature of posts.**

**Sharing personal experience**

- Senders share how medicine shortages affect them (personally or professionally) or their loved ones.
- Post contains a phrase such as “My medicine is not available,” or “This medicine is unavailable, and I have to assure patients they can safely use an alternative.”

**Sharing emotions**

- Senders express their emotional responses to a medicine shortage.
- These posts use words expressing an emotion (eg, angry or sad) or figures of speech, such as sarcasm.
- Post contains a phrase such as “What a nightmare!”

**Call to action**

- Senders call on influential people or institutions to act.
- These posts use a direct mention (@) or a hashtag (#) combined with the name of the person or institution.
- Post contains a phrase such as “@Min of Health.”

**Providing information**

- Senders post objective information to a wider audience.
- Post contains phrase such as “There will be a temporary shortage of medicine X.”

**Seeking medicine**

- Senders try to find the medicine in shortage.
- Post contains a phrase such as “Does anyone happen to have some medicine left?”

**Request for advice**

- Senders ask for general or specific advice.
- Post contains a phrase such as “What should I do now?”

**Explaining or advising someone**

- Senders explain or advise an individual.
- Post contains a phrase such as “@Username: I want to refer you to this link for more information.”

**Amplifying message**

- Senders aim to reach a significant number of people.
- Post contains a phrase such as “This medicine is almost entirely unavailable in the Netherlands.”

All data used in this study were collected according to Twitter’s terms of use and were publicly available at the time of collection and analysis. Research on data from social media, such as Twitter, makes tracing direct quotations back to the user easy. Users expect that results from Twitter are anonymized [33]. Thus, reporting direct quotations is questionable. Modifying post text conflicts with Twitter display requirements prevents altering posts [34]. Thus, we did not quote individual posts and only paraphrased examples of posts in Textbox 1.

Statistical analysis was performed using SPSS (version 28; IBM Corp), and other data analyses were performed using Microsoft Office 365 Excel.

### Ethical Considerations

This analysis did not receive approval from an institutional research board. Analysis of large bodies of text written in social media such as the Twitter social network is generally not considered “human subjects research” [35], and therefore, ethical approval was not sought.

## Results

### Selection of the Posts

The search for Dutch posts using the names of the 341 medicines in shortage retrieved 47,193 posts, 40,104 of which were unique. After selecting posts about medicine shortages by searching for relevant keywords, 2644 posts remained. After assessing the posts, we excluded 1909 posts because they mentioned keywords in a different context or medicine as an alternative to overcome a shortage of another medicine. The remaining posts (n=735) were analyzed.

### Mentioned on the Twitter Social Network or Not

Of the 341 medicines in shortage, 102 (29.9%) were mentioned on Twitter during the shortage within the specified timeframe (±30 days). Among these 102 medicines, the median number of posts per medicine was 2 (IQR 1-5; range 1-50).

**Figure 1:**
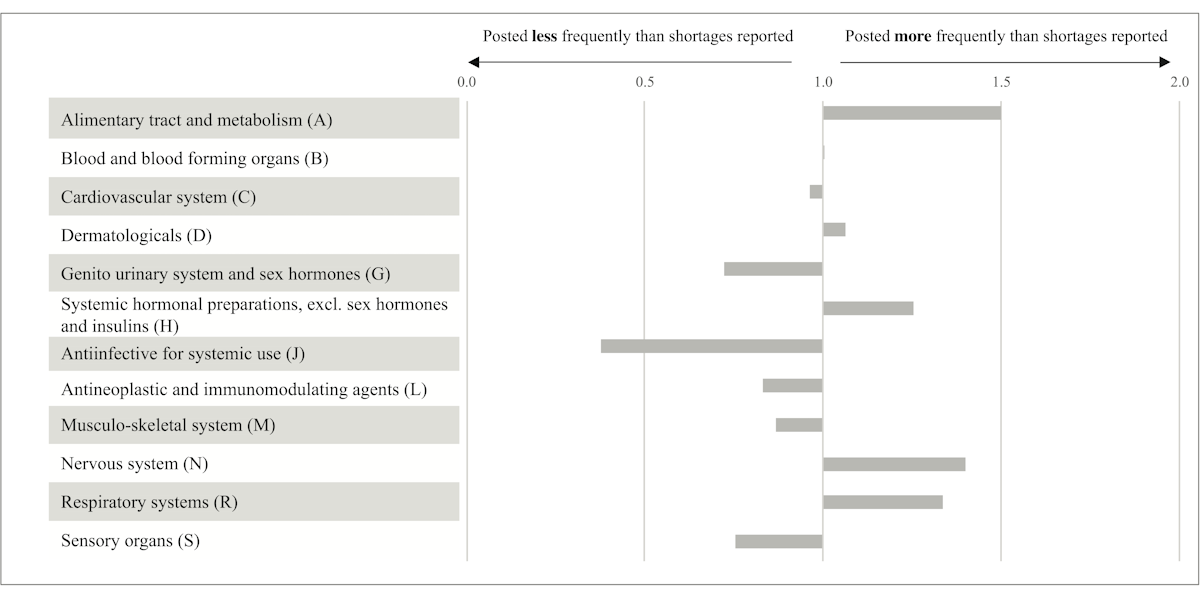
The number of medicines in shortage mentioned in posts compared to the number of medicines in shortage (per ATC1 class). At 1.0, the proportion of posts for this ATC class is equal to the proportion of published shortages. ATC: Anatomical Therapeutic Chemical; ATC1: Anatomical Therapeutic Chemical class on the first level.

In comparing the number of medicines mentioned in posts to the number of medicines in shortage, the alimentary tract and metabolism (ATC class A) and the nervous system (ATC class N) were mentioned 1.5 and 1.4 times more often on Twitter, respectively, than expected, based on the observed number of shortages. Anti-infectives for systemic use (ATC class J) were mentioned 2.5 times less than expected (Figure 1; underlying data can be found in Multimedia Appendix 2). Differences were borderline significant (Fisher test Monte Carlo with a 99% CI: 21,390; *P*=.048, 99% CI .042-.054).

Shortages of oral preparations were mentioned more often on Twitter (n=69, 67.6% vs n=140, 58.6%), and parenteral preparations were mentioned less often on Twitter (n=11, 10.8% vs n=56, 23.4%), but the differences between routes of administration were—also borderline—not statistically significant (Fisher exact test=11,779; *P*=.051; Table 1).

Shortages mentioned on Twitter more often involved therapeutic substitution as a possible alternative product, according to KNMP Farmanco (n=18, 22.8% versus n=21, 13.6% among shortages not found on Twitter). This difference was not statistically significant (Fisher exact test=3441; *P*=.16; Table 1). Shortages mentioned on Twitter affected a higher number of patients than those that were not mentioned (median 31,760, IQR 8735-86,583 versus 4636, IQR 630-22,134; median test: *P*<.001). The median age of patients using medicines in shortage reported on Twitter was 60 (IQR 50-68) years; for medicines not mentioned on Twitter, it was 61 (IQR 48-69) years. The age difference was insignificant (median test: *P*=.68). Finally, shortages on Twitter had a significantly longer duration (149 days) than shortages not mentioned on Twitter (104 days; median test: *P*<.001).

**Table 1:** Characteristics of medicines in shortage and patients affected (mentioned on Twitter vs not mentioned on Twitter).

				Number of medicine shortages		
				Mentioned on Twitter (n=102)		Not mentioned on Twitter (n=239)
**Product characteristics, n (%)**						
	**Medicine group (ATC1^a^)**					
		Alimentary tract and metabolism (A)	13 (12.7)		16 (6.7)	
		Blood and blood forming organs (B)	3 (2.9)		7 (2.9)	
		Cardiovascular system (C)	15 (14.7)		37 (15.5)	
		Dermatologicals (D)	7 (6.9)		15 (6.3)	
		Genito urinary system and sex hormones (G)	5 (4.9)		18 (7.5)	
		Systemic hormonal preparations, excl. sex hormones and insulins (H)	3 (2.9)		5 (2.1)	
		Anti-infective for systemic use (J)	4 (3.9)		31 (13.0)	
		Antineoplastic and immunomodulating agents (L)	5 (4.9)		15 (6.3)	
		Musculo-skeletal system (M)	6 (5.9)		17 (7.1)	
		Nervous system (N)	28 (27.5)		39 (16.3)	
		Antiparasitic products, insecticides and repellents (P)	0 (0.0)		3 (1.3)	
		Respiratory system (R)	8 (7.8)		12 (5.0)	
		Sensory organs (S)	5 (4.9)		17 (7.1)	
		Various (V)	0 (0.0)		7 (2.9)	
	**Route of administration**					
		Oral	69 (67.6)		140 (58.6)	
		Parenteral	11 (10.8)		56 (23.4)	
		Nasal or inhalation	5 (4.9)		9 (3.8)	
		Cutaneous	8 (7.8)		14 (5.9)	
		Rectal	0 (0.0)		1 (0.4)	
		Ocular	4 (3.9)		15 (6.3)	
		Other	5 (4.9)		4 (1.7)	
**Patient impact, n (%)**						
	**Alternative product^b^**					
		Generic substitution	59 (74.7)		130 (84.4)	
		Therapeutic substitution	18 (22.8)		21 (13.6)	
		Import	2 (2.5)		3 (1.9)	
	Number of patients affected, median (IQR)		31,760 (8735-86,583)		4636 (630-22,134)	
	Age of patients affected (years), median (IQR)		60 (50-68)		61 (48-69)	
	Shortage duration^c^ (days), median (IQR)		149 (82-248)		104 (58-190)	

^a^ATC1: Anatomical Therapeutic Chemical class on the first level.

^b^n=275 because not all advised alternatives could be traced.

^c^For permanent shortages, the end date was set at December 31, 2019, to calculate the duration.

### Timing of Posts

For 18 of the 102 shortages mentioned on Twitter, posts were published before or simultaneous to the publication by KNMP Farmanco and 4 were posted more than 14 days earlier (Table 2). Of the 18 shortages, 8 (44%) concerned medicines for the nervous system (N). These shortages early mentioned on Twitter, were mainly posted by patients (n=11, 61%).

**Table 2:** Shortages mentioned on Twitter before publication by KNMP^a^ Farmanco.

Active substance	Medicine group (ATC1^b^)	Stakeholder	Days between first publication by Twitter versus KNMP Farmanco, n
Cabergoline	Genitourinary system and sex hormones (G)	Health care professionals	–27
Salmeterol and fluticasone	Respiratory systems (R)	Health care professionals	–26
Acamprosate	Nervous system (N)	Patients	–24
Olanzapine	Nervous system (N)	Health care professionals	–15
Naproxen	Musculo-skeletal system (M)	Patients	–13
Mirtazapine	Nervous system (N)	Patients	–13
Dexamfetamine	Nervous system (N)	Health care professionals	–12
Aciclovir	Anti-infective for systemic use (J)	Unknown	–11
Disulfiram	Nervous system (N)	Health care professionals	–11
Colestyramine	Cardiovascular system (C)	Patients	–7
Paracetamol	Nervous system (N)	Patients	–6
Mycophenolic acid	Antineoplastic and immunomodulating agents (L)	Patients	–5
Citalopram	Nervous system (N)	Patients	–5
Acetazolamide	Sensory organs (S)	Patients	–3
Tamoxifen	Antineoplastic and immunomodulating agents (L)	Patients	–2
Vancomycin	Anti-infective for systemic use (J)	Health care professionals	–1
Pyridostigmine	Nervous system (N)	Patients	0
Famotidine	Alimentary tract and metabolism (A)	Patients	1

^a^KNMP: Royal Dutch Pharmacists Association.

^b^ATC1: Anatomical Therapeutic Chemical class on the first level.

**Figure 2:**
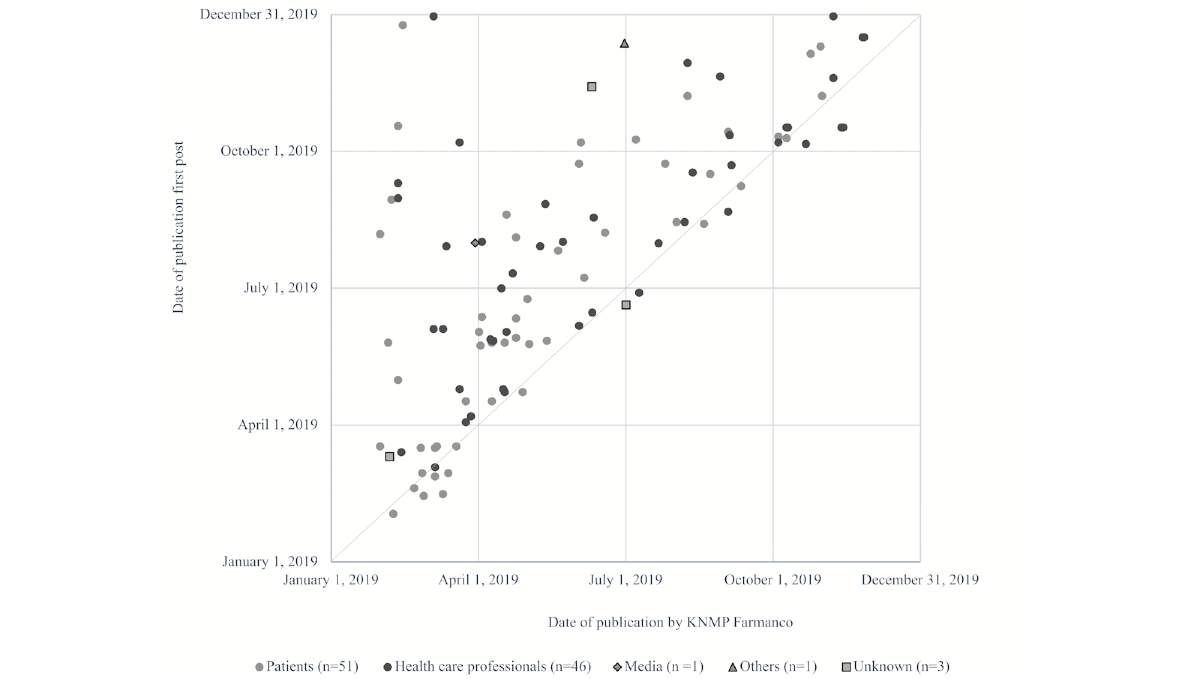
Date of publication of a medicine shortage (n=102) by KNMP Farmanco versus the first time mentioned on Twitter, per stakeholder. KNMP: Royal Dutch Pharmacists Association.

**Figure 3:**
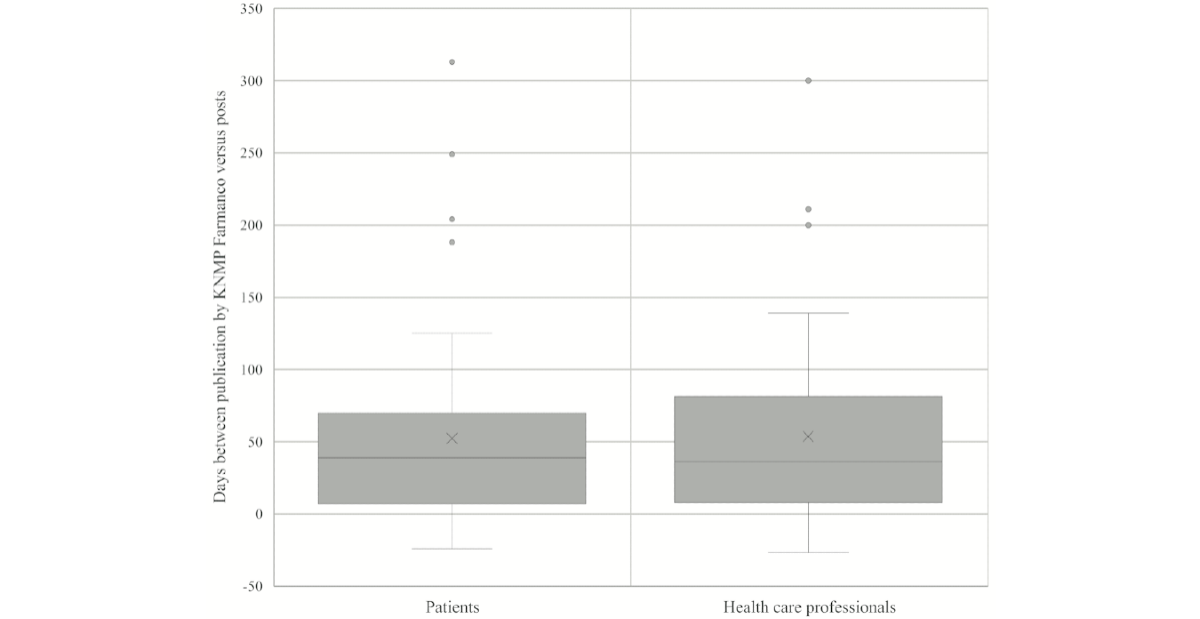
Days between the publication of a medicine shortage by KNMP Farmanco versus the first time mentioned on Twitter (patients and health care professionals). KNMP: Royal Dutch Pharmacists Association.

For the 102 shortages with matched posts, the median difference between publication by KNMP Farmanco and the first published post was 37 (IQR 7-81) days, meaning that KNMP Farmanco identified the shortages earlier than Twitter (Figure 2). There was no difference in the median delay of posts published by patients (39, IQR 7-69 days) or health care professionals (36, IQR 8-81 days; median test: *P*=.840; Figure 3). Posts about medicines for the nervous system (N) were published with a median delay of 30 days. Medicines for the alimentary tract and metabolism (A) and the cardiovascular system (C) were posted later (median of 48 and 67 days, respectively; Multimedia Appendix 3).

### Content Analysis

The first posts on the 102 shortages were published by patients (n=51, 50%), health care professionals (n=46, 45.1%), the media (n=1, 1%), others (n=1, 1%), and unknown users (n=3, 2.9%). We identified 8 categories of posts (Table 3). Sharing personal experience (n=44, 43.1%) was the main category. Emotions, such as anger, frustration, and despair, were regularly shared (n=21, 20.6%), or calls to action were made (n=13, 12.7%). A similar pattern is visible in all 735 posts on medicine shortages (Multimedia Appendix 4).

**Table 3:** Nature of first posts on medicine shortages per stakeholder (n=102).

Nature	Patients, n	Health care professionals, n	Media, n	Others, n	Unknown, n
Sharing personal experience	18	26	N/A	N/A	N/A
Sharing emotions	12	9	N/A	N/A	N/A
Call to action	6	5	N/A	N/A	2
Providing information	4	3	N/A	1	N/A
Seeking for medicine	3	N/A^a^	N/A	N/A	N/A
Request for advice	5	N/A	N/A	N/A	N/A
Explaining or advising someone	3	3	N/A	N/A	1
Amplifying message	N/A	N/A	1	N/A	N/A

^a^N/A: not applicable.

## Discussion

### Principal Findings

Our study showed that a routine analysis of Twitter data is not well suited as an early warning system for medicine shortages. Of the 341 medicine shortages, only 18 (5.3%) were mentioned on Twitter prior to or simultaneous to publication by KNMP Farmanco. Only 4 (1.2%) shortages were mentioned on Twitter more than 14 days before publication by KNMP Farmanco. Although we observed differences, we could not conclusively relate the presence or absence on Twitter to a disease area or route of administration of the medicine in shortage and, thus, to certain patient groups.

Two factors may explain why only a limited number of medicine shortages are mentioned on Twitter simultaneously or prior to publication by KNMP Farmanco. First, Twitter does not represent the general population. In 2019, 2.8 million people (16.2% of the population of 17.3 million) used Twitter in the Netherlands, and the usage varied among age groups [36]. Patients affected by the medicine shortages (regardless of being mentioned on Twitter) were a median age of 60 years, an age group that uses Twitter less than other age groups [36]. Second, most terms searched on Twitter, such as the names of medicines, are specific. Spelling errors and references to more general terms, for instance, heart pills and birth control, are not detected, resulting in a limited number of posts. A recent study on early warnings for signals of adverse events and the pharmacovigilance of medicines also detected only a limited number of signals [28], probably for the same reason. Nevertheless, using Twitter as an early warning system for COVID-19 outbreaks was found to be an effective approach [26,37]. This search strategy contained more general terms.

Thus, we expected shortages with a high impact on patients to be mentioned on Twitter more often. We analyzed 2 elements that are core drivers of patient impact—alternative products and the number of patients affected [38]. The availability of an alternative product with the same substance and route of administration (generic substitution) was expected to have less patient impact than that of an alternative product with a different substance (therapeutic substitution). For example, a shortage of aciclovir eye ointment had a high patient impact since no eye drop or ointment was available to treat an eye infection caused by the herpes simplex virus, making therapeutic substitution necessary. Nevertheless, this shortage was not mentioned on Twitter. Although shortages solved by therapeutic substitution were mentioned more often on Twitter than shortages solved by generic substitution, this difference was not statistically significant. In contrast, the number of patients affected, another core driver, showed a significant difference. If more people were affected, it was likelier that the medicine shortage would be mentioned on Twitter.

Even though shared personal experiences comprised the main content of the analyzed posts, over 1 in 5 posts expressed emotions. As expected, patients showed anger, frustration, and despair, and health care professionals did as well. This finding demonstrated that medicine shortages are not solely an administrative problem; they also affect patients and health care professionals personally.

Detecting medicine shortages as early as possible creates time to find solutions to mitigate the shortages’ impact on patients. However, identifying medicine shortages and subsequent data sharing may also lead to undesirable reactions such as panic buying and hoarding. Harnessing the dividends of information sharing requires standardized procedures, close collaboration and exchange of information between stakeholders, as well as accurate timing [8].

The major strength of this study is the direct comparison between a public catalog of medicine shortages and posts on medicine shortages on social media platforms such as Twitter. We could establish all the characteristics of the medicine shortages published by KNMP Farmanco and all those of Dutch posts on these shortages. To our knowledge, this study is the first to investigate the suitability of social media as an early warning system for medicine shortages. Detecting medicine shortages as early as possible is expected to create time to find solutions to mitigate the shortages’ impact on patients. Thus, social media platforms should be researched as possible sources of information. Moreover, many countries outside of Europe do not yet publish national catalogs on medicine shortages and may be investigating information sources for detecting medicine shortages. Unfortunately, though, Twitter does not serve as an early warning system in the Netherlands.

During analyses, we read the emotional responses and perspectives of patients and health care professionals that have not been described in research. The impact of medicine shortages on patients has been limited to qualitative research on a small number of patients. For research on a larger population of patients and health care professionals that is affected by shortages, Twitter may provide the opportunity for future qualitative research [39]. At the time of research, Twitter was a social media platform with free access for users and nonusers. It is unclear if this access-policy will remain. A change in policy, terms of use, availability of viable alternatives, or public opinion has proven to result in a change for users [40]. Therefore, for future research other social media platforms should also be considered. Like signal detection in current pharmacovigilance systems, Twitter cannot be used in isolation, but it can be used in combination with traditional systems for early signal detection because it can provide a holistic profile [27].

### Limitations

Our study has several limitations related to search strategies. First, we used a selection of shortages that KNMP Farmanco published, but we did not use potential shortages that people identified on Twitter that KNMP Farmanco did not publish. Second, we used the active pharmaceutical ingredient’s name and the brand name’s first word to identify relevant posts. As previously mentioned, our search strategy did not allow us to capture posts about medicines that contained a spelling error in the substance or brand name or posts that only mentioned a more general description of the medicine (for example, “heart pills” or “birth control”). Missing posts with these possible errors or using general terms may mean that specific shortages may have been reported earlier on Twitter than we currently found. However, since we were aiming to assess the role of posts on Twitter for routine analysis, our approach resembles this potential use.

### Comparison With Prior Work

We expected lively discussions on Twitter on medicine shortages because there have been increasingly more medicine shortages in the Netherlands in recent years [31,41]. This phenomenon [42] and specific shortages have been discussed in the national news. For instance, a national shortage of contraceptive pills in late 2018 resulted in many news articles [43] and national television reports [44]. This media attention was related to that on Twitter, and we hypothesized that Twitter might serve as a new but still underused data source for identifying medicine shortages. However, the results did not confirm this hypothesis. Only 735 (1.6%) of the 47,193 posts on the medicines in shortage discussed the shortage, while the other 46,458 (98.4%) were concerned with other aspects of the medicines. Two-thirds of the shortages were not even discussed on Twitter.

### Conclusions

Twitter cannot be considered an early warning system for medicine shortages, since only 102 of the 341 medicines in shortage were mentioned on Twitter. Of these 102, only 18 were mentioned on Twitter prior to or simultaneous to publication by KNMP Farmanco and only 4 shortages were mentioned on Twitter more than 14 days before publication by KNMP Farmanco. Posts on Twitter tend to echo known information rather than spread new information. However, Twitter has the potential added value of providing a broader view of the personal experiences of patients and health care professionals with medicine shortages. Twitter, or potentially any other social media platform, provides the opportunity for future qualitative research in the increasingly important field of medicine shortages that investigates how a larger population of patients is affected by shortages.

## Appendix

Multimedia Appendix 1 Key words potentially indicating a medicine shortage.

Multimedia Appendix 2 Number of medicines in shortage mentioned in posts compared to number of medicines in shortage (per ATC1 class). ATC: Anatomical Therapeutic Chemical.

Multimedia Appendix 3 Number of days between publication by KNMP Farmanco versus social network Twitter (per ATC1 class). ATC: Anatomical Therapeutic Chemical; KNMP: Royal Dutch Pharmacists Association.

Multimedia Appendix 4 Content analysis of posts on medicine shortages in 2019 (n=735).

## Abbreviations

ATC: Anatomical Therapeutic Chemical
KNMP: Royal Dutch Pharmacists Association
MAH: marketing authorization holder
SFK: Dutch Foundation for Pharmaceutical Statistics

## Data Availability

The data set on medicine shortages not publicly available, since data are related to specific products and manufacturers, which is confidential information. The data set on posts is not publicly available due to privacy or ethical restrictions but are available from the corresponding author upon reasonable request. Also, Twitter offers the service Twitter’s API (application programming interfaces) [39]. Data on the number of users are not publicly available since it is third-party data. These data belong to the Dutch Foundation for Pharmaceutical Statistics (SFK). The SFK welcomes applications from researchers for access to data. Application forms for data access and further information are available [45].
