# Supplementary material for: Identifying Medicine Shortages With the Twitter Social Network: Retrospective Observational Study
Source: J Med Internet Res. 2024 Aug 6;26:e51317. doi: 10.2196/51317 (PMC11336501; doi:10.2196/51317)
Supplement: Multimedia Appendix 1 [file jmir_v26i1e51317_app1.pdf]

MULTIMEDIA APPENDIX 1 - KEY WORDS POTENTIALLY INDICATING A MEDICINE SHORTAGE

DUTCH	ENGLISH TRANSLATION
Ander merk	Other brand
Aanwezig	Present
Alternatieven	Alternatives
Beschikbaar	Available
Bijna op	Almost out of
Disponibel	Accessible
Gebrek	Deficiency
Geleverd	Supplied
Handelsvergunning	Marketing authorization
Leverbaar	Available
Leverdatum	Delivery date
Leveren	To supply
Levering	Supply
Manco	Shortcoming
Niet aanwezig	Not present
Ontbreek	Lack
Ontbreken	Lack of
Ontoereikend	Inadequate
Onvoldoende	Insufficient
Schaars	Scarce
Te krijgen	Available
Tekort	Shortage
Ter beschikking	Available
Uit de handel	Discontinued
Verkrijgbaar	Accessible
Verkrijgen	To obtain
Voorhanden	Obtainable
Voorraad	Stock
Voorradig	Stocked
Zoektocht	Search
