# Supplementary material for: Identifying Medicine Shortages With the Twitter Social Network: Retrospective Observational Study
Source: J Med Internet Res. 2024 Aug 6;26:e51317. doi: 10.2196/51317 (PMC11336501; doi:10.2196/51317)
Supplement: Multimedia Appendix 2 [file jmir_v26i1e51317_app2.pdf]

MULTIMEDIA APPENDIX 2 - NUMBER OF MEDICINES IN SHORTAGE MENTIONED IN POSTS COMPARED TO NUMBER OF MEDICINES IN SHORTAGE (PER ATC1 CLASS)

ATC class on the first level (ATC1)	Shortages; n (%)	Shortages posted on Twitter; n (%)	Comparison
Alimentary tract and metabolism (A)	29 (8.5)	13 (12.7)	1.5
Blood and blood forming organs (B)	10 (2.9)	3 (2.9)	1.0
Cardiovascular system (C)	52 (15.2)	15 (14.7)	1.0
Dermatologicals (D)	22 (6.5)	7 (6.9)	1.1
Genito urinary system and sex hormones (G)	23 (6.7)	5 (4.9)	0.7
Systemic hormonal preparations, excl. sex hormones and insulins (H)	8 (2.3)	3 (2.9)	1.3
Antiinfective for systemic use (J)	35 (10.3)	4 (3.9)	0.4
Antineoplastic and immunomodulating agents (L)	20 (5.9)	5 (4.9)	0.8
Musculo-skeletal system (M)	23 (6.7)	6 (5.9)	0.9
Nervous system (N)	67 (19.6)	28 (27.5)	1.4
Respiratory systems (R)	20 (5.9)	8 (7.8)	1.3
Sensory organs (S)	22 (6.5)	5 (4.9)	0.8
