# Supplementary material for: Identifying Medicine Shortages With the Twitter Social Network: Retrospective Observational Study
Source: J Med Internet Res. 2024 Aug 6;26:e51317. doi: 10.2196/51317 (PMC11336501; doi:10.2196/51317)
Supplement: Multimedia Appendix 3 [file jmir_v26i1e51317_app3.pdf]

MULTIMEDIA APPENDIX 3 - NUMBER OF DAYS BETWEEN PUBLICATION BY KNMP FARMANCO VERSUS SOCIAL NETWORK TWITTER (PER ATC1)

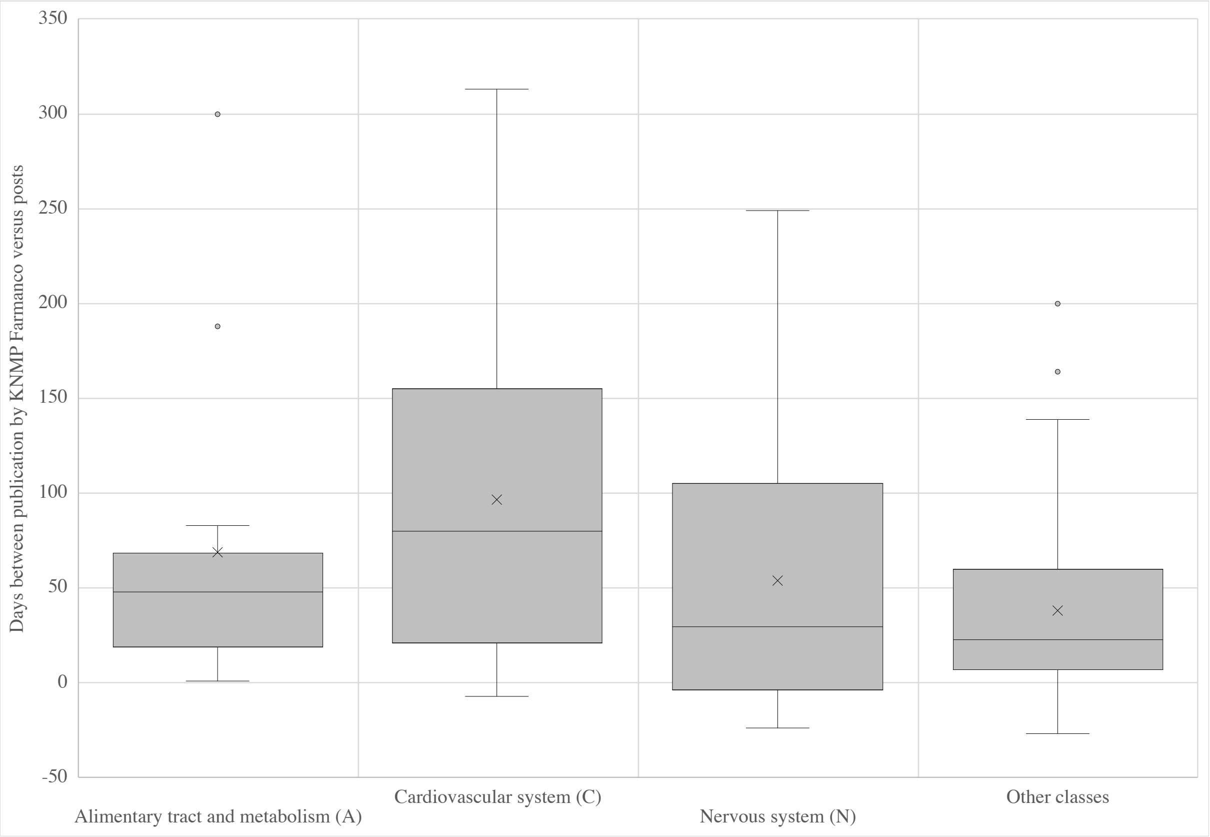

	median	IQR1	IQR3	Number of posts
**Alimentary tract and metabolism (A)**	48	30	54	13
**Cardiovascular system (C)**	80	31	139	15
**Nervous system (N)**	30	-1	103	28
**Other classes**	23	7	57	46
