# Supplementary material for: Identifying Medicine Shortages With the Twitter Social Network: Retrospective Observational Study
Source: J Med Internet Res. 2024 Aug 6;26:e51317. doi: 10.2196/51317 (PMC11336501; doi:10.2196/51317)
Supplement: Multimedia Appendix 4 [file jmir_v26i1e51317_app4.pdf]

MULTIMEDIA APPENDIX 4 - CONTENT ANALYSIS OF POSTS ON MEDICINE SHORTAGES IN 2019 (N = 735)

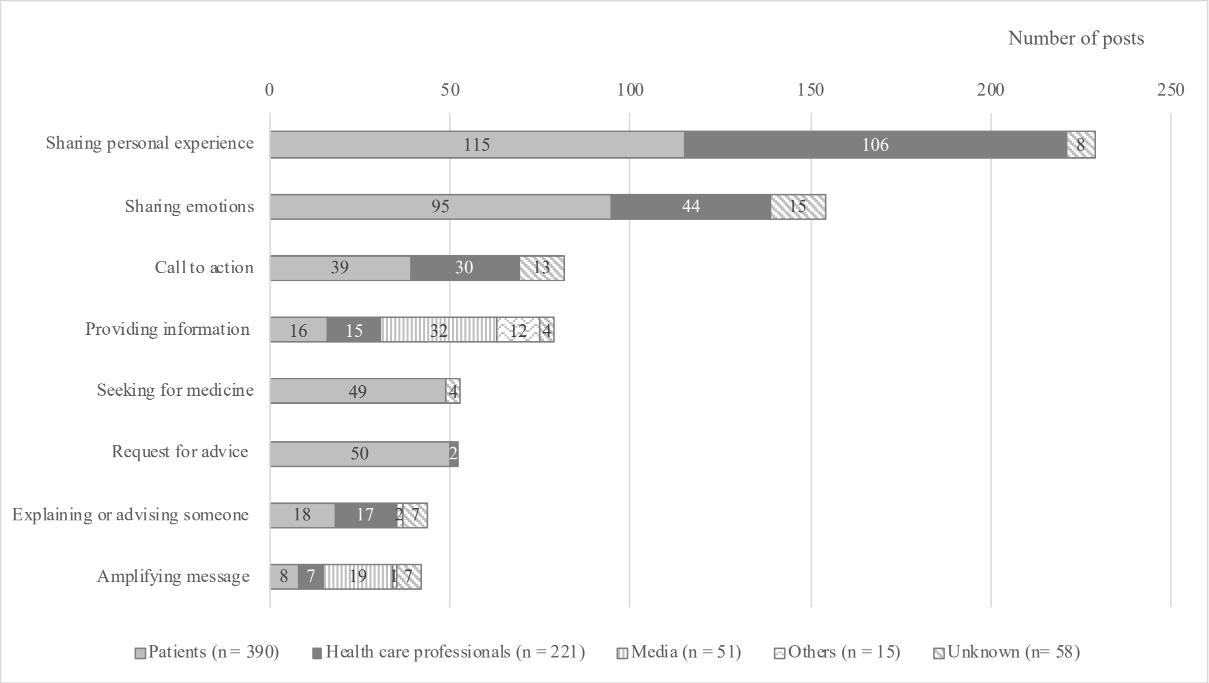

	PATIENTS	HEALTH CARE PROFESSIONALS	MEDIA	OTHERS	UNKNOWN
**SHARING PERSONAL EXPERIENCE**	115	106			8
**SHARING EMOTIONS**	95	44			15
**CALL TO ACTION**	39	30			13
**PROVIDING INFORMATION**	16	15	32	12	4
**SEEKING FOR MEDICINE**	49				4
**QUESTION/REQUEST FOR ADVICE**	50	2			
**EXPLAINING OR ADVISING SOMEONE**	18	17		2	7
**AMPLIFYING MESSAGE**	8	7	19	1	7
